# The Effects of Hospital Noise and Noise Sensitivity on Patient`s Comfort, Annoyance, and Intention to Leave

**DOI:** 10.12688/f1000research.167974.2

**Published:** 2026-02-28

**Authors:** Milad Abbasi, Mahdi Sharifpour, Mahtab Mohammadi, Yasin Manoochehri, Tahereh Eskandari

**Affiliations:** 1 Saveh university of medical sciences, Saveh, 9898989898, Iran; 2 Islamic Azad University Sanandaj Branch, Sanandaj, Kurdistan Province, Iran; 3 Department of Occupational Health Engineering, Iran university of medical sciences, Tehran, 01010101010, Iran

**Keywords:** Hospital noise, Noise Sensitivity, Noise Annoyance, Acoustic Comfort, Intention to Leave

## Abstract

**Introduction:**

Hospitals are intended to serve as healing environments; however, they are frequently characterized by high levels of environmental noise pollution that can contradict their therapeutic purpose. This cross-sectional study aimed to investigate the complex relationships among hospital noise pollution, individual noise sensitivity, and acoustic comfort, noise annoyance, and intention to leave the hospital.

**Methods:**

This descriptive-analytical cross-sectional study was conducted in 2024 at a public hospital in Saveh, Iran. A stratified random sampling method with proportional allocation was used to select a sample of 226 hospitalized adult patients. Objective day-evening-night noise levels (L _den_) were measured over 24 hours, while subjective data on noise sensitivity, acoustic comfort, noise annoyance, and intention to leave the hospital were collected using standardized questionnaires. Bayesian Network (BN) modeling, a probabilistic graphical approach for examining complex dependencies, was applied in combination with delta-p sensitivity analysis to quantify the direct and joint effects of noise exposure and noise sensitivity on patient outcomes. Continuous variables were categorized using percentile cut-offs: low (<P25), moderate (P25–P75), and high (>P75).

**Results:**

The mean L _den_ in the studied hospital was found to be 57.95 dB (±6.61). The Bayesian Network analysis revealed that under conditions of high level L _den_, the probability of high annoyance, low acoustic comfort and high intention to leave increased by 12.4%, 6.3% and 5%, respectively. Under conditions of high-Level Sensitivity, the probability of these variables increased by 9.1%, 6.2% and 4.7%, respectively. While these two variables are at high level, the most substantial positive variations occurred in high annoyance, low acoustic comfort and high intention to leave, with increases of 26.1%, 13.1% and 10.6%.

**Conclusion:**

Noise levels in the hospital exceed international standards, negatively affecting acoustic comfort, increasing annoyance, and influencing individuals’ intent to leave. Personal noise sensitivity further intensifies these effects.

## 1. Introduction

Hospitals are intended to serve as healing environments, yet they are often characterized by excessive levels of environmental noise that can contradict their therapeutic purpose. Sources of hospital noise are numerous and varied, ranging from medical equipment, ventilation systems, and staff activities, to patient movement and visitor interactions. ^1, 2^ In high-density hospital settings, noise levels frequently exceed internationally recommended thresholds, potentially interfering with both patient recovery and staff performance. ^3^ The World Health Organization suggests that noise levels in hospital wards should not exceed 35 dB during the day and 30 dB at night. However, many studies have shown that these levels are consistently surpassed, often reaching peaks above 85 dB. ^4, 5^

Excessive noise in healthcare facilities is more than a simple environmental nuisance—it can have complex consequences on physiological, psychological, and behavioral health. Research has demonstrated associations between prolonged exposure to noise and hearing impairments, risk of cancer, elevated heart rates, blood pressure irregularities, increased cortisol levels, and sleep disturbances in patients. ^6– 8^ On a psychological level, noise contributes to anxiety, reduced performance, stress, and emotional distress, complicating recovery trajectories. ^9– 11^ For healthcare providers, the effects are similarly detrimental, leading to higher levels of occupational burnout, fatigue, and even intention to resign. ^12, 13^ Such outcomes underline the imperative need to understand and mitigate the impact of hospital noise exposure.

Among patients, individual differences in noise sensitivity may moderate their responses to environmental soundscapes. Noise sensitivity is a stable personality trait that reflects how strongly a person reacts to noise, independent of the objective sound intensity. ^10^ Highly noise-sensitive individuals are more likely to experience discomfort and perceive sound as more disturbing than others, which may amplify their annoyance and overall dissatisfaction with the hospital experience. ^14^ In clinical settings, noise-sensitive patients might display lower pain thresholds, poorer sleep quality, and greater susceptibility to noise-induced stress.

One critical dimension in understanding patients’ reactions to noise is acoustic comfort—a concept referring to the subjective perception of the acoustic environment as pleasant, tolerable, and non-disruptive. While often discussed in architectural and environmental psychology literature, acoustic comfort has recently attracted attention in hospital research due to its connection to patient satisfaction and healing outcomes. ^15^ Factors influencing acoustic comfort include background noise levels, variability, predictability, and the perceived control over the auditory environment. ^6^ Poor acoustic comfort can lead to reduced trust in medical care, increased anxiety, and decreased willingness to remain in the facility.

Relatedly, noise annoyance—defined as a subjective negative reaction to noise—is one of the most reported emotional responses among hospitalized individuals. Annoyance can arise from both the intensity and the meaning attributed to noise, with many patients perceiving certain sounds (e.g., alarms, staff conversations) as intrusive or inappropriate in a care context. ^1^ Chronic annoyance is not merely an emotional state but has been linked to increased health risks and behavioral outcomes, such as reduced adherence to treatment, complaints, and negative evaluations of care. ^9^

Perhaps one of the most understudied yet consequential behavioral outcomes in hospital noise research is intention to leave, which refers to the patient’s conscious consideration or decision to discharge prematurely due to discomfort or dissatisfaction. While extensively studied among healthcare staff in relation to burnout and workplace environment, ^12^ little is known about how environmental noise might affect patients’ intentions to leave. This is particularly relevant given that early discharge—if not medically indicated—can negatively affect health outcomes and impose further burdens on healthcare systems. Despite growing awareness of hospital noise pollution, existing research has predominantly focused on staff-related outcomes or examined noise as an isolated environmental factor, with limited empirical attention to how individual noise sensitivity interacts with acoustic conditions to shape patient-centered outcomes such as acoustic comfort, noise annoyance, and behavioral intentions. ^16, 17^ This gap is particularly pronounced in low- and middle-income countries—including Iran—where healthcare facilities often rely on generalized international guidelines (e.g., WHO, EPA) in the absence of enforceable national standards tailored to local contexts, resulting in scarce region-specific data to inform acoustic design and noise reduction policies. ^5^

Addressing these gaps requires a multidimensional framework that integrates environmental, psychological, and behavioral domains. Conventional regression approaches are ill-equipped to capture the complex, non-linear, and interdependent relationships inherent in such a framework. To overcome this methodological limitation, the present study applies Bayesian Network (BN) modeling—a probabilistic graphical approach that explicitly models conditional dependencies, visualizes causal structures, and quantifies both direct and joint effects—enabling a more nuanced examination of how noise exposure and noise sensitivity synergistically influence patient outcomes. Accordingly, this study was designed to examine the complex relationships among hospital noise pollution, noise sensitivity, and key patient outcomes including acoustic comfort, noise annoyance, and intention to leave, thereby contributing to the evidence base for acoustically mindful hospital design and policy in settings where such data are urgently needed.

## 2. Methodology

### 2.1 Study design and context

This descriptive-analytical cross-sectional study with a basic-applied orientation was conducted in 2024 across a public hospital in Saveh, Iran, with the aim of investigating how environmental noise pollution and individual differences in noise sensitivity impact patients’ acoustic comfort, noise-related annoyance, and intention to leave the hospital. The hospital setting, with its complex combination of medical equipment, staff movement, and patient activities, provided a real-world context in which environmental acoustics play a crucial role in shaping patient experience.

### 2.2 Participants and sampling strategy

The minimum required sample size was determined using G*Power 3.1 software, assuming a medium effect size (0.3), α = 0.05, and a statistical power of 0.95. Based on these parameters, a minimum sample of 178 participants was deemed necessary. To account for potential attrition, this number was increased by 15%, yielding a final sample of 205 participants—the minimum acceptable number for sufficient statistical power and adequate generalizability. During data collection, a total of 226 eligible patients were recruited and participated voluntarily. This larger achieved sample strengthens the generalizability of the findings and is fully consistent with the sampling strategy, as no upper limit was imposed on participation.

The study population consisted of hospitalized adult patients who had stayed for a minimum of 48 hours in one of the active hospital wards, including internal medicine, general surgery, and coronary care unit (CCU) department. Eligible participants were those who were 18 years of age or older, fully conscious, capable of verbal communication, and clinically stable during the time of data collection. Patients were excluded if they had been transferred to intensive care units, experienced acute medical deterioration, or provided incomplete questionnaire responses.

Sampling was conducted using a stratified random sampling method with proportional allocation. Initially, a comprehensive list of all hospital wards was prepared, and a proportionate number of patients was randomly selected from each ward based on its occupancy size. Within each ward, participants were selected randomly and voluntarily from different patient rooms. This approach ensured a representative distribution of the sample across the hospital’s various departments, thereby increasing the external validity of the findings.

To further enhance data reliability and contextual accuracy, data collection was carried out through interviewer-administered questionnaires. Researchers were physically present at the patients’ bedside, assisting participants in completing the questionnaires. This hands-on approach not only improved response accuracy but also enabled researchers to better understand the patients’ subjective experiences with hospital noise in real time. In accordance with the Declaration of Helsinki, ethical approval for this study was granted by the Medical Ethics Committee of Saveh University of Medical Sciences (Ethics Code: IR.SAVEHUMS.REC.1403.039). All procedures were conducted in full compliance with the approved ethical guidelines. Prior to participation, all individuals received comprehensive information regarding the study’s objectives, methodology, and potential risks. Written informed consent was obtained from all participants, affirming their voluntary participation.

### 2.3 Objective measurement of day-evening-night noise levels (L _den_)

Environmental noise data were collected over a three-month period from 10 November 2024 to 10 February 2025, across multiple active hospital wards under the jurisdiction of Saveh University of Medical Sciences. To comprehensively capture acoustic variability throughout the day and night, equivalent continuous sound pressure levels were recorded in eight distinct time intervals: 07:00–10:00, 10:00–13:00, 13:00–16:00, 16:00–19:00, 19:00–22:00, 22:00–01:00, 01:00–04:00, and 04:00–07:00. These cycles were systematically rotated across weekdays to ensure that the acoustic conditions were representative of both weekdays (Saturday to Thursday in Iran) and weekends (Friday). These intervals were later aggregated into three standardized periods for analysis—day (07:00–19:00), evening (19:00–23:00), and night (23:00–07:00). Noise exposure levels were assessed as equivalent continuous sound pressure levels (Leq) over consecutive 15-minute intervals, with each 15-minute Leq measurement serving as a proxy for the corresponding three-hour timeframe. These 15-minute Leq values were aggregated to derive cumulative noise levels for the morning, afternoon, and nighttime periods. Over a 24-hour monitoring cycle, individual participants contributed 120 minutes of sampled data. Across the entire study, this methodology yielded a comprehensive dataset exceeding 24,600 minutes of noise measurements, collected between October 2024, and February 2025. To calculate the weighted 24-hour equivalent noise level, the Level day-evening-night (L _den_) values corresponding to the day, evening, and night periods were combined using the following standard formula, which accounts for increased human sensitivity during non-daytime hours ^18^:

$$L_{\mathrm{den}}=10.\log_{10}\;\left(\frac{1}{24}\left(12.10^{\frac{L_{\mathrm{day}}}{10}}+4.10^{\frac{L_{\text{evening}}+5}{10}}+8.10^{\frac{L_{\text{night}}+10}{10}}\right)\right)$$

In this formula, L _day_, L _evening_, and L _night_ represent the measured equivalent noise levels during the morning, evening, and night intervals, respectively. The weighting factors (i.e., +5 dB for evening and +10 dB for night) reflect internationally accepted corrections for human sensitivity to noise during these periods, as outlined by the World Health Organization and ISO acoustic standards.

Noise exposure was assessed using a B&K Sound Level Meter Analyzer, model 2250, which was calibrated before each measurement session using a TES 1356 calibrator at 1000 Hz and 94 dB. The sound level meter was operated in the “slow” response mode and adjusted to an appropriate range to capture real-time acoustic fluctuations in the hospital environment. While the measurement protocol generally followed principles outlined in ISO 9612:2009, adjustments were made to suit clinical conditions. Specifically, the microphone was positioned at the typical auditory height of a recumbent patient, facing dominant noise sources within each ward to ensure ecological validity.

### 2.4 Questionnaires

Data collection was performed using a structured questionnaire pack including demographic data and four psychometrically validated instruments:

*Background and demographic information*

Basic demographic and background data were collected using a researcher-designed form. This section of the instrument gathered information on the participants’ age, gender, marital status, and the hospital ward in which they were admitted. These variables were used to provide a descriptive profile of the sample and to contextualize the interpretation of the study findings.

*Weinstein Noise Sensitivity Scale (WNSS)*

The WNSS is a 21-item scale developed to assess individual noise sensitivity, an internal trait influencing reactions to acoustic stimuli. Items are rated on a 6-point Likert scale (0 = “completely agree” to 5 = “completely disagree”), yielding scores between 0 and 105, with higher scores indicating greater sensitivity. ^19^ The Persian version, validated by Alimohammadi et al., demonstrated good internal consistency (Cronbach’s α = 0.78) and has been used in various Iranian populations. ^20^

*Acoustic comfort scale*

Perceived acoustic comfort was measured through a 7-point semantic differential scale ranging from 1 (very uncomfortable) to 7 (very comfortable). This instrument captures the subjective evaluation of soundscapes, a construct increasingly relevant in hospital design and healthcare experience research. ^21^

*Visual Analogue Scale (VAS)*

This study utilized a 100-mm Visual Analogue Scale (VAS) to measure noise annoyance and intention to leave the hospital, with endpoints anchored at 0 (“not at all”) and 100 (“extremely”). The VAS is widely recognized for its sensitivity and precision in capturing subjective experiences in healthcare settings, offering distinct advantages over categorical scales, including greater measurement granularity and reduced susceptibility to ceiling effects. ^22, 23^ The validity of using VAS for noise-related outcomes in hospitals has been demonstrated in prior research. Sayılan et al. (2021), for example, used the VAS to assess patient responses to varying noise levels in intensive care units, reporting statistically significant differences across acoustic conditions (p < 0.01). This finding underscores the responsiveness of the VAS to environmental factors within clinical contexts. ^24^ Given its methodological strengths—including ease of administration, enhanced sensitivity, and minimal cognitive burden—the VAS serves as an optimal tool for evaluating noise-related perceptions and behavioral intentions in inpatient populations.

*Visual analogue scale for noise annoyance*

Noise annoyance was measured using a 100-mm Visual Analogue Scale (VAS). Participants were asked the following question:

“How much do the sounds and noises in the hospital bother (annoy) you?”

They responded by marking a point on a horizontal line, where 0 indicated “not at all” and 100 indicated “extremely bothersome”. Higher scores reflected greater perceived annoyance due to hospital noise.

*Visual analogue scale for intention to leave the hospital*

Intention to leave the hospital was assessed using a single-item VAS. Participants were asked:

“How much does the noise in the hospital make you want to leave or change your room?”

Responses were marked on a 100-mm line anchored at 0 (“no desire to leave”) and 100 (“very strong desire to leave”). Higher scores indicated a stronger inclination to leave or avoid the hospital environment due to noise exposure.

### 2.5 Statistical analysis

Subsequent to the completion of data acquisition, a preliminary phase of statistical analysis was executed using SPSS software, version 27. This encompassed the generation of descriptive statistics, including measures of central tendency (mean, median, mode) and dispersion (standard deviation, variance, range), as detailed in Reference [ 25]. These initial analyses provided a foundational understanding of the data distribution and variability, crucial for subsequent modeling.

Building upon this descriptive foundation, Bayesian Networks (BNs), a class of probabilistic graphical models pioneered by Pearl, ^26^ were deployed to explore the complex interrelationships within the data. BNs, by their nature, represent systems as directed acyclic graphs (DAGs), where nodes symbolize variables, and edges signify probabilistic dependencies. This framework explicitly delineates cause-effect relationships, enabling the visualization and quantification of how changes in one variable propagate through the system to influence others. Their ability to integrate heterogeneous data types, including both quantitative and qualitative variables, and to model complex interactions and outcomes, while simultaneously facilitating the exploration of trade-offs, positions BNs as particularly advantageous for modeling intricate causal systems. Moreover, BNs demonstrate resilience in handling data originating from diverse sources and are adept at managing datasets with missing values through probabilistic inference. The inherent causal graphical structure of BNs, characterized by conditional dependencies, promotes accessibility, allowing for the construction of models without requiring extensive technical modeling expertise and facilitating comprehension by non-technical stakeholders, a feature of substantial practical utility. ^26, 27^

The BN topology (nodes and directed links) was defined to reflect the study structure and was derived from the variables/domains captured by the questionnaires used in this study. The BN was then used for probabilistic inference (evidence propagation) to quantify interdependencies among nodes and to examine how changing the state of one or more nodes updates the probability distributions of other variables. Conditional Probability Tables (CPTs) were subsequently estimated from the observed study data using GeNIe.

The construction and analysis of the Bayesian network were carried out using GeNIe software, version 2.0. Following the development of the BN graphical structure, which involved defining nodes and edges based on domain knowledge and data relationships, Conditional Probability Tables (CPTs) were generated. ^28^ CPTs quantify the conditional probabilities of each node given its parent nodes, providing a complete probabilistic specification of the network. To determine the relative importance of individual variables within the network, a delta-p sensitivity analysis was conducted. ^29, 30^ This analysis involved systematically varying the states of input nodes and observing the resulting changes in the probabilities of target nodes. In intuitive terms, Δp quantifies how much the probability of each state of a target node (low, moderate, high) changes after setting evidence on an input node to a specific state (low, moderate, or high), while keeping the BN structure constant. For each evidence scenario, Δp for a given target state was computed as the updated probability of that state minus its baseline probability prior to setting evidence (updated − baseline). Larger |Δp| values indicate a stronger influence on that target state, and the sign indicates whether the probability of that state increases (+) or decreases (−).

Continuous variables were discretized prior to Bayesian Network modeling using sample-based percentile thresholds. Values below the 25th percentile (P25) were defined as low, values between the 25th and 75th percentiles (P25–P75) as moderate, and values above the 75th percentile (P75) as high. The same categorization rule was applied consistently across all continuous variables included in the model.

## 3. Results

A total of 226 hospitalized patients participated in the study, with near-equal gender distribution (49.6% male, 50.4% female). The majority were married (73.0%), and approximately half were admitted to internal medicine wards (42.2%), followed by general surgery (37.2%) and the coronary care unit (18.6%). Detailed demographic characteristics are presented in Table 1.

**Table 1.** Demographic characteristics of the study participants.

Variable	Category	Frequency	Percent	Cumulative
Gender	Male	112	49.6	49.6
	Female	114	50.4	100.0
	Total	205	100.0	-
Marital Status	Single	61	27.0	27.0
	Married	165	73.0	100.0
	Total	205	100.0	-
Hospital Ward	Internal Medicine	100	42.2	42.2
	General Surgery	84	37.2	81.4
	Coronary Care Unit	42	18.6	100.0
	Total	226	100.0	-

The mean ± SD of the dimensions of abovementioned variables is presented in Table 2.

**Table 2.** Descriptive statistics and categorical distributions of study variables.

Variable	Level	Frequency	Percent	Total measured Mean	Total measured SD
L _den_	Low	70	31	57.95	06.61
	Moderate	87	38.5		
	High	69	30.5		
Noise Sensitivity	Low	55	24.8	42.57	18.16
	Moderate	113	50.9		
	High	54	24.3		
Noise Annoyance	Low	79	35	38.46	17.07
	Moderate	74	32.7		
	High	73	32.3		
Acoustic Comfort	Low	94	25.2	04.73	01.45
	Moderate	63	36		
	High	69	38.7		
Intention to leave	Low	58	27.5	26.06	11.78
	Moderate	87	38.5		
	High	81	35.8		

Note: Lden = day-evening-night weighted equivalent noise level. Mean and SD values represent the original continuous measurements for each variable prior to categorization. Lden, Noise Sensitivity, Noise Annoyance, Acoustic Comfort, Intention to.

**Figure 1.**
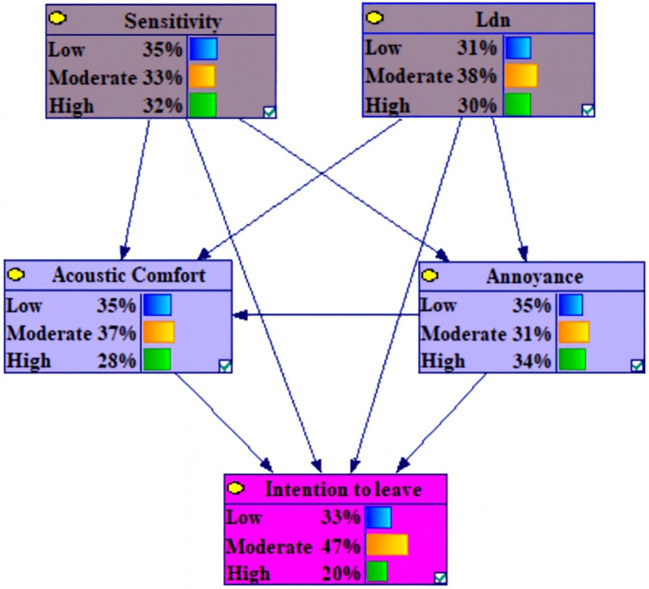
The dependencies among the marginal probabilities of the studied variables based on the Bayesian network model. Nodes represent variables; directed edges (arrows) indicate conditional dependencies. Lden (day-evening-night noise level) and Noise Sensitivity are exogenous predictors. Noise Annoyance and Acoustic Comfort are positioned as intermediate nodes in the probabilistic pathways from Lden and Sensitivity to Intention to Leave, consistent with a mediating structure.

The dependencies among the marginal probabilities of the studied variables based on the Bayesian network model is shown in the Figure 1.

Table 3 illustrates the marginal probability distribution, derived from the Conditional Probability Tables (CPT), for the examined variables. The CPTs quantify the associations among the variables.

**Table 3.** Marginal probability distributions of study variables derived from Bayesian Network model.

Variable	Level	Marginal probability distribution
L _den_	Low	0.310
	Moderate	0.385
	High	0.305
Sensitivity	Low	0.350
	Moderate	0.327
	High	0.323
Annoyance	Low	0.346
	Moderate	0.313
	High	0.341
Acoustic Comfort	Low	0.350
	Moderate	0.371
	High	0.279
Intention to leave	Low	0.326
	Moderate	0.470
	High	0.204

Note: Values are rounded consistently based on the original Bayesian Network outputs; therefore, each probability distribution sums to 100%.

Model updating was performed by instantiating the target node as evidence, leading to a revision of all variable probability distributions within the Bayesian Network. The updated probabilities are detailed in Table 4.

**Table 4.** Updated probability distributions of outcome variables under five evidence scenarios.

Variable	Level	L _den_ (High 100%)	Noise Sensitivity (High 100%)	L _den_ (High 100%) and Noise Sensitivity (Low 100%)	L _den_ (Low 100%) and Noise Sensitivity (High 100%)	L _den_ and Noise Sensitivity (High 100%)
L _den_	Low	0	0.310	0	1	0
	Moderate	0	0.385	0	0	0
	High	1	0.305	1	0	1
Noise Sensitivity	Low	0.350	0	1	0	0
	Moderate	0.327	0	0	0	0
	High	0.323	1	0	1	1
Noise Annoyance	Low	0.252	0.250	0.350	0.350	0.150
	Moderate	0.284	0.320	0.300	0.350	0.250
	High	0.464	0.430	0.350	0.300	0.600
Acoustic Comfort	Low	0.414	0.410	0.350	0.350	0.481
	Moderate	0.358	0.360	0.380	0.380	0.319
	High	0.228	0.230	0.270	0.270	0.200
Intention to leave	Low	0.254	0.254	0.320	0.325	0.180
	Moderate	0.492	0.494	0.460	0.455	0.510
	High	0.254	0.252	0.220	0.220	0.310

Note: Baseline probabilities represent the marginal distribution from the Bayesian network prior to evidence instantiation. Values are rounded consistently based on the original Bayesian Network outputs; therefore, each probability distribution sums to 100%.

**Figure 2.**
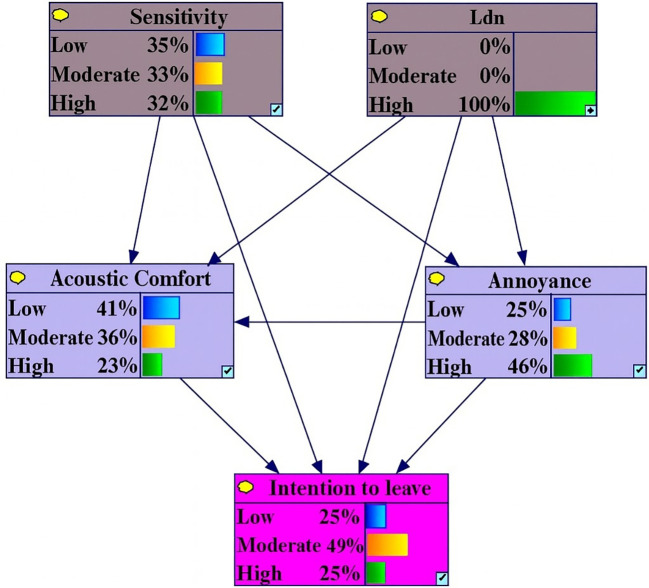
Sensitivity analysis on high L _den._ Delta-p sensitivity analysis results under evidence scenario: Noise exposure = 100% High. Bars represent percentage point changes in the probability of each outcome variable state relative to baseline marginal probabilities. Positive values (right-directed bars) indicate increased probability; negative values (left-directed bars) indicate decreased probability.

Under conditions of high Level L _den_, the probability of high annoyance, low acoustic comfort and intention to leave variables increased by 12.4%, 6.3% and 5%, respectively. In contrast, the probabilities of low annoyance, low intention to leave and high acoustic comfort decreased by 9.4%, 7.4% and 5.1% ( Figure 2).

**Figure 3.**
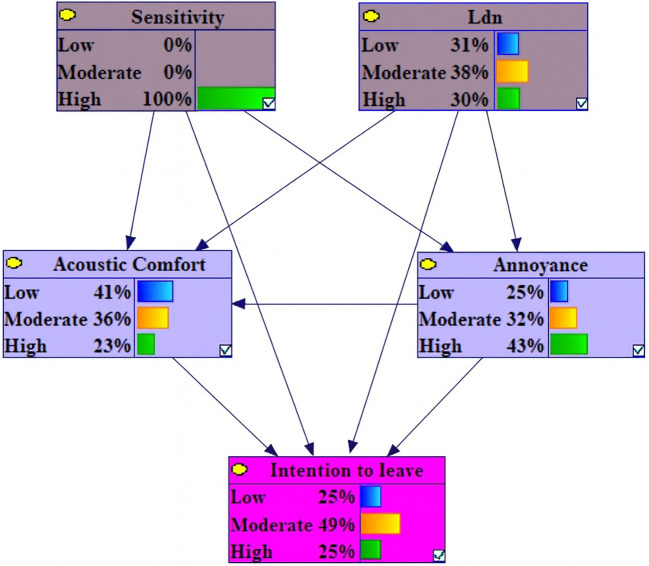
Sensitivity analysis on high sensitivity. Delta-p sensitivity analysis results under evidence scenario: Noise Sensitivity = 100% High. Bars represent percentage point changes in the probability of each outcome variable state relative to baseline marginal probabilities. Positive values (right-directed bars) indicate increased probability; negative values (left-directed bars) indicate decreased probability.

Under conditions of high-Level Sensitivity, the probability of high annoyance, low acoustic comfort and high intention to leave variables increased by 9.1%, 6.2 and 4.7%, respectively. In contrast, the probabilities of low annoyance, low intention to leave and high acoustic comfort decreased by 9.6%, 7.2% and 5.2% ( Figure 3).

**Figure 4.**
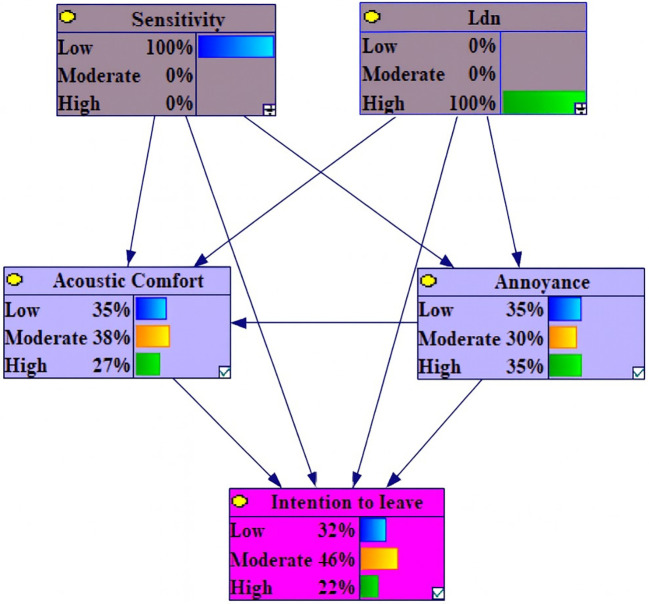
Sensitivity analysis on high Lden and low sensitivity. Delta-p sensitivity analysis results under evidence scenario: Lden = 100% High AND Noise Sensitivity = 100% Low. Bars represent percentage point changes in outcome probabilities relative to baseline.

Among the variables under conditions of 100% High Level L _den_ and 100% Low Sensitivity, the changes were observed in: a 1.8% increase in high intention to leave, a 1.1% increase in moderate acoustic comfort and low annoyance, a 1.5% decrease in moderate intention to leave and a 1.3% decrease in low annoyance ( Figure 4). It is notable that the extent of changes in this section is negligible.

**Figure 5.**
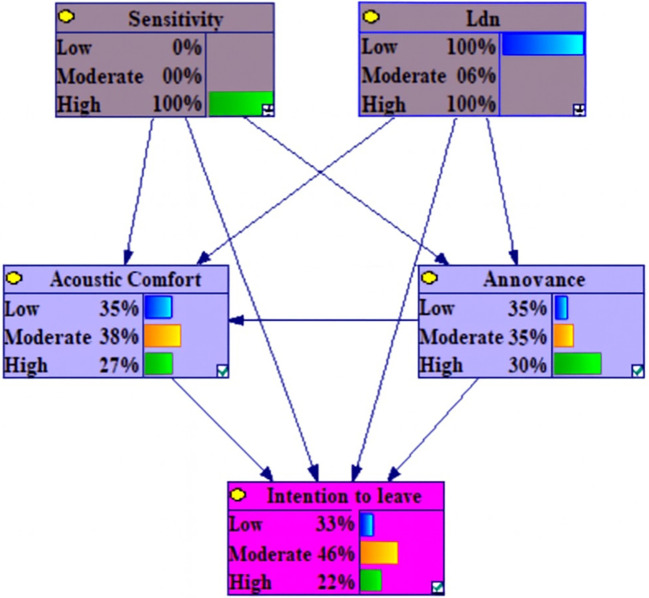
Sensitivity analysis on low L _den_ and high sensitivity. Delta-p sensitivity analysis results under evidence scenario: Lden = 100% Low AND Noise Sensitivity = 100% High. Bars represent percentage point changes in outcome probabilities relative to baseline.

Concerning the variables under the stipulated conditions of 100% Low Level L _den_ and 100% High sensitivity, the most prominent augmentations were evidenced in moderate annoyance, high intention to leave and moderate acoustic comfort, quantified at 3.7%, 1.5%, and 1.1%, respectively. Conversely, diminutions of 3.9%, 3%, and 1.5% were observed in the values of high annoyance, low acoustic comfort and moderate intention to leave ( Figure 5).

**Figure 6.**
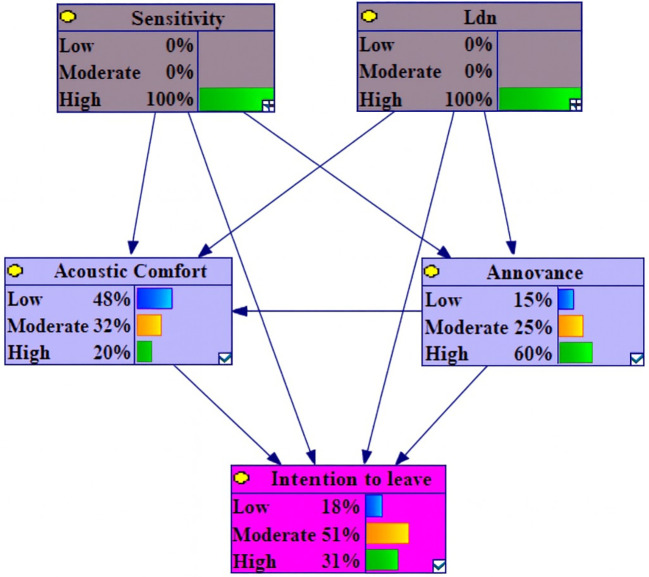
Sensitivity analysis on high L _den_ and sensitivity. Delta-p sensitivity analysis results under evidence scenario: Lden = 100% High AND Noise Sensitivity = 100% High. Bars represent percentage point changes in outcome probabilities relative to baseline.

Under conditions of 100% High Level L _den_ and Sensitivity, analysis of change variables revealed that the most substantial positive variations occurred in high annoyance, low acoustic comfort and high intention to leave, with increases of 26.1%, 13.1% and 10.6%. Conversely, a corresponding analysis indicated decreases of 19.6%, 14.3% and 10.5% in low annoyance and intention to leave ( Figure 6).

The sensitivity analysis of the studied variables is reported in Table 5, with a positive sign indicating an increase and a negative sign indicating a decrease.

**Table 5.** Delta-p sensitivity analysis: percentage point changes in outcome probabilities under five evidence scenarios.

Variable	Level	L _den_ (High 100%)	Sensitivity (High 100%)	L _den_ (High 100%) and Sensitivity (Low 100%)	L _den_ (Low 100%) and Sensitivity (High 100%)	L _den_ and Sensitivity (High 100%)
Lden	Low	-	0	-	-	-
	Moderate	-	0	-	-	-
	High	-	0	-	-	-
Sensitivity	Low	0	-	-	-	-
	Moderate	0	-	-	-	-
	High	0	-	-	-	-
Annoyance	Low	-9.4 %	-9.6 %	+0.4 %	+0.4 %	-19.6 %
	Moderate	+3 %	+0.6 %	-1.3 %	+3.7%	-6.3 %
	High	+12.4 %	+ 9.1%	+ 1.1%	- 3.9%	+26.1
Acoustic Comfort	Low	+ 6.3 %	+ 6.2%	0%	- 3%	+ 13.1%
	Moderate	-1.3 %	- 1.1%	+1.1%	+1.1%	- 5.2 %
	High	-5.1%	-5.2%	-1.1%	-0.8%	- 7.8%
Intention to leave	Low	-7.2%	-7.2%	- 0.4%	- 0.1 %	-14.3%
	Moderate	+2.2%	+2.4%	- 1.5 %	- 1.5 %	+3.7 %
	High	+ 5%	+4.7%	+ 1.8%	+ 1.5%	+ 10.6 %

Note: Values represent the difference (in percentage points) between the posterior probability under each evidence scenario and the baseline marginal probability ( Table 4).

## 4. Discussion

The present study elucidates the complex interplay between hospital noise pollution, noise sensitivity, and critical patient outcomes, including acoustic comfort, noise annoyance, and intention to leave. Our findings reveal several noteworthy patterns that both corroborate and extend existing literature in healthcare acoustics. The observed mean L den level of 57.95 dB (±6.61) substantially exceeds WHO recommendations (35 dB daytime/30 dB nighttime). This finding mirrors recent global studies. Nyembwe et al. (2023) documented comparable noise levels (55–72 dB) in ICUs across Congolese hospitals, and Amoatey et al. (2022) reported 24-hour averages of 63.5 dB in Omani healthcare facilities. ^4, 5^

Bayesian Network analysis demonstrated that high noise sensitivity amplified the negative effects of noise exposure. When both factors were present simultaneously, the probability of high noise annoyance increased by 26.1%. This finding aligns with emerging neurophysiological evidence from Zhou et al. (2020), whose fMRI studies revealed heightened amygdala activation in noise-sensitive individuals exposed to hospital sounds. ^6^ The observed moderated mediation pattern provides empirical support for the Stressor-Sensitivity Model proposed by Gong et al. (2022). This model posits that individual differences in sensory processing modulate environmental stress responses. ^9^ Noise annoyance has long been recognized as one of the primary effects of noise exposure across various environments. It is also closely correlated with individual noise sensitivity. In other words, noise annoyance is influenced by two sets of variables: noise exposure, which acts as an external factor, and noise sensitivity, which is an individual-specific characteristic. The results of the present study indicate that both of these factors contribute to increased annoyance among patients. Moreover, when both variables were set at their highest levels in the model, they demonstrated a synergistic effect, leading to a significantly greater increase in perceived annoyance. The literature has shown that noise annoyance acts as a mediator of the various health and psychological outcomes of noise exposure. ^31^ Numerous studies have indicated that individuals who are highly sensitive to noise experience greater annoyance when exposed to environmental noise, which in turn is associated with more adverse health outcomes. In this study, it was found that noise annoyance increased under all conditions — whether due to high noise exposure or heightened individual sensitivity to noise. This increase in annoyance was accompanied by a corresponding rise in the intention to leave the hospital. Thus, both noise exposure and noise sensitivity influence the intention to leave the hospital through pathways that may include direct effects as well as indirect effects via noise annoyance. However, formal statistical mediation testing was not conducted in this study. In all cases, increases in either noise levels or sensitivity led to higher intentions to leave hospital. It has been reported that annoying stimuli such as noise, can increase individual arousal and provide a motivating reason to leave the environment. ^32^ Therefore, it can be concluded that in hospital settings, noise annoyance may lead patients to decide to leave the facility prematurely, before completing their prescribed course of hospitalization.

Acoustic comfort is a subjective evaluation of the sound environment, influenced by both external acoustic stimuli and individual psychological predispositions such as noise sensitivity. Patients with heightened noise sensitivity tend to appraise hospital soundscapes more negatively, reporting lower levels of acoustic comfort even under moderate noise conditions. ^6^ This discomfort may stem from sensory overload, heightened vigilance, or perceived loss of control over the environment. In the present study, patients with high noise sensitivity demonstrated a pronounced decrease in acoustic comfort, particularly when ambient noise levels were also elevated. Reduced acoustic comfort, in turn, was associated with an increased intention to leave the hospital. This pattern is consistent with a mediating role for acoustic comfort, though formal mediation analysis was not performed. Studies suggest that when patients feel aurally overwhelmed, their sense of well-being declines. This decline leads to emotional fatigue and decreased willingness to remain in the hospital. ^9^ Thus, noise sensitivity and environmental exposure jointly reduce acoustic comfort. This reduction may, in turn, contribute to patients' decisions to leave the care environment prematurely—a pathway suggestive of mediation. Beyond its effects on comfort and annoyance, the interaction between noise exposure and individual sensitivity may directly influence patients’ behavioral responses, including the decision to leave the hospital early. In our model, high L _den_ and elevated noise sensitivity increased the probability of moderate and high intention to leave by more than 3.7% and 10.6%, respectively. This behavior may be driven by psychological mechanisms such as avoidance coping or perceived threat, wherein patients interpret persistent noise as a signal of low-quality care or lack of control. Premature hospital discharge has been associated with adverse outcomes, including incomplete treatment, higher readmission rates, and reduced patient satisfaction. ^14^ While such departures are typically multifactorial, environmental discomfort—particularly in the form of uncontrolled noise—may act as a powerful yet under-recognized contributor.

According to the results presented in Table 5, noise annoyance exhibits greater variability compared to acoustic comfort, primarily due to fluctuations in both noise sensitivity and exposure levels. This finding highlights the significance of noise annoyance as a key factor within hospital environments. Numerous studies have demonstrated that noise annoyance has a substantial impact on various aspects of health, including the elevation of stress hormone levels. ^9^ Furthermore, it can contribute to increased anxiety and depressive symptoms in individuals. ^9, 33^ Considering that patients are often already in a compromised state of health, noise annoyance may exacerbate their medical conditions by heightening stress, anxiety, and depression, potentially leading them to leave the hospital prematurely—before completing the prescribed treatment process. In summary, noise annoyance, as the most prominent consequence of noise exposure and heightened noise sensitivity, can give rise to a wide range of adverse health outcomes. ^33^

## 5. Limitations

While this study provides valuable insights into the relationships between hospital noise pollution, individual noise sensitivity, and key patient outcomes, several limitations should be considered. First, the study was conducted in a single public hospital, which may limit the generalizability of the findings to other healthcare settings, particularly private institutions or those with different infrastructural and organizational characteristics. Second, the cross-sectional design restricts the ability to establish causal or temporal relationships between environmental noise exposure and patients’ psychological or behavioral responses. Third, although ward type was accounted for, other potentially influential clinical factors—such as pain levels, medication use, or individual health conditions—were not controlled. Forth, this study focused exclusively on acoustic environmental factors and did not measure other physical environmental parameters (e.g., thermal comfort, lighting, air quality, privacy) that may also influence patients' intention to leave. Moreover, the use of L _den_ as the sole noise metric, while practical and standardized, may have failed to capture more granular temporal fluctuations in noise exposure that could influence patient responses. Lastly, the use of a single-item Visual Analogue Scale to measure intention to leave, while appropriate for this unidimensional and context-specific construct, does not permit assessment of internal consistency and may not capture the full complexity of patients' decision-making processes. More broadly, reliance on self-reported measures introduces the possibility of response bias, despite the use of validated instruments. Development and validation of multi-item instruments for patient voluntary departure intention attributable to environmental factors, as well as incorporation of objective behavioral indicators (e.g., actual against-medical-advice discharge rates, room transfer requests), are important priorities for future research.

## 6. Policy and design implications

This persistent non-compliance with international standards across diverse healthcare systems suggests a systemic failure in noise control implementation that transcends geographical boundaries. Therefore, mitigating hospital noise and addressing the needs of noise-sensitive individuals is not only a matter of comfort, but also a determinant of patient retention and treatment adherence. Both noise exposure and noise sensitivity influence the intention to leave the hospital through direct and indirect pathways, with noise annoyance serving as an intermediate variable. Noise annoyance, as the most prominent consequence of noise exposure and heightened sensitivity, can give rise to a wide range of adverse health outcomes. Accordingly, addressing noise pollution—particularly through targeted interventions for noise-sensitive individuals and improved acoustic design—should be viewed as an essential dimension of quality healthcare delivery.

## 7. Conclusion

Hospital soundscapes are active components of the care environment, not merely ambient features. This study demonstrates that noise exposure and individual noise sensitivity synergistically increase annoyance, reduce acoustic comfort, and elevate patients' intention to leave. The findings position acoustic comfort and noise annoyance as intermediate variables in these pathways. Addressing noise pollution—particularly through targeted interventions for noise-sensitive individuals and improved acoustic design—is therefore an essential dimension of quality healthcare delivery.

## Ethics approval and consent to participate

In accordance with the Declaration of Helsinki, ethical approval for this study was granted by the Medical Ethics Committee of Saveh University of Medical Sciences (Ethics Code: IR.SAVEHUMS.REC.1403.039). All procedures were conducted in full compliance with the approved ethical guidelines.

## Data Availability

**Figshare:** The Effects of Hospital Noise Pollution and Noise Sensitivity on Patient’s Acoustic Comfort, Noise Annoyance, and Intention to Leave, https://doi.org/10.6084/m9.figshare.30373360.v1. ^34^ The project contains the following underlying data:
•
Hospital_Noise_Study_Data.sav: (A structured data file containing all anonymized patient responses and calculated scores for acoustic comfort, noise annoyance, noise sensitivity, and intention to leave.) Data are available under the terms of the Creative Commons Attribution 4.0 International license (CC-BY 4.0).
