# Supplementary material for: The Effects of Hospital Noise and Noise Sensitivity on Patient`s Comfort, Annoyance, and Intention to Leave
Source: F1000Res. 2026 Feb 28;14:1250. Originally published 2025 Nov 14. [Version 2] doi: 10.12688/f1000research.167974.2 (PMC13019034; doi:10.12688/f1000research.167974.2)
Supplement: Peer review history

# Reviewer report: Reviewer response for version 2

**DOI:** 10.5256/f1000research.196838.r463664

**Published:** 2026-03-27

**Authors:** Sanjay Kumar

**Affiliations:** 1 University of Nebraska-Lincoln, Omaha, USA

The revised manuscript titled “The Effects of Hospital Noise and Noise Sensitivity on Patient’s Comfort, Annoyance, and Intention to Leave” demonstrates substantial improvement and now meets the standards for indexing. The authors have addressed prior reviewer comments thoroughly, particularly by clarifying the study design, strengthening the theoretical framework, and providing more robust statistical analyses. The discussion has been significantly enhanced, offering clearer interpretation of the findings and stronger connections to existing literature, which reinforces the study’s relevance to healthcare environment management and patient-centered care. Additionally, the revisions improve the manuscript’s coherence, methodological transparency, and practical implications, especially regarding noise mitigation strategies in hospital settings. Overall, the study offers meaningful and timely insights into how environmental factors influence patient experience and behavioral intentions, making it a valuable contribution to the field. I therefore recommend acceptance in its current form.

Is the work clearly and accurately presented and does it cite the current literature?

Yes

If applicable, is the statistical analysis and its interpretation appropriate?

Yes

Are all the source data underlying the results available to ensure full reproducibility?

Yes

Is the study design appropriate and is the work technically sound?

Yes

Are the conclusions drawn adequately supported by the results?

Yes

Are sufficient details of methods and analysis provided to allow replication by others?

Yes

Reviewer Expertise:

Hospital noise, Environmental Noise, Metamaterials, Urban Built environment

I confirm that I have read this submission and believe that I have an appropriate level of expertise to confirm that it is of an acceptable scientific standard.

# Reviewer report: Reviewer response for version 2

**DOI:** 10.5256/f1000research.196838.r463665

**Published:** 2026-03-25

**Authors:** Domenico De Salvio

**Affiliations:** 1 University of Bologna, Bologna, Italy

The paper investigates an important topic in healthcare environments: the relationship among noise pollution, noise sensitivity, and patient annoyance. The study is interesting, well written, and well described. The reproducibility of the method is quite ensured. While the idea of using a Bayesian Network is helpful for linking objective and subjective data, several methodological issues arise. Following my points:

 - Sampling only 15 minutes to representatively describe 3 hours of noise is strongly limiting. The noise environment in hospitals is dynamic, driven by various sources with distinct spectral and temporal features that deeply affect noise annoyance assessment. Also, impulsive noises can be highly intrusive.

 - I don't understand why the authors stated that the monitoring they performed doesn't allow a statistical analysis of percentile levels. 

 - Noise monitoring is the only objective data underlying the analysis of the context. No information about the interval time or the features extracted is provided. 

 - L_den is scarcely representative of the annoyance in such a dynamic context. All impulsive events are squeezed into a single number with no information about the nature of the disturbing noise. This metric is usually used in the context of transportation noise, where noise spectra are well-defined.

 - Also, low frequencies play a key-role in annoyance. No mention has been made about it.

 - In section 2.5, it is written, "This framework explicitly delineates cause-effect relationships, enabling the visualization and quantification of how changes in one variable propagate through the system to influence others". This is misleading because a cause-and-effect relationship implies causality, and a single Bayesian Network cannot assess causality within a given context. It provides conditional probabilities, not causalities.

 - Continue variables like VAS score are forced to be categorized in "Low", "Moderate", and "High". This is fundamental for the use of CPT, but it strongly squeezes the data's variance. Thus, the study's sensitivity in describing patients' annoyance and intention to leave is deeply affected.

 Since this is the second round of the review, my review focuses on the extension of Section 5 - Limitations. Here, all the concerns listed before should be addressed and described in the paper. While this section analyzes the limits of the work, it should also describe potential ways to deepen it. Particularly, the use of advanced methods to analyze noise data is carefully described in:

 Refer to reference no. 1-4

 I think that adding a broader, more conscious section on the study's limitations and highlighting its potential for future development can make the reader more aware of the work's importance. Also, providing appropriate literature that enables in-depth physiological and technical analysis for scholars is fundamental. 

Is the work clearly and accurately presented and does it cite the current literature?

Partly

If applicable, is the statistical analysis and its interpretation appropriate?

Partly

Are all the source data underlying the results available to ensure full reproducibility?

No source data required

Is the study design appropriate and is the work technically sound?

Partly

Are the conclusions drawn adequately supported by the results?

Partly

Are sufficient details of methods and analysis provided to allow replication by others?

Yes

Reviewer Expertise:

Noise monitoring and analysis

I confirm that I have read this submission and believe that I have an appropriate level of expertise to confirm that it is of an acceptable scientific standard, however I have significant reservations, as outlined above.

## References

[1] : Relating clustered noise data to hospital patient satisfaction. *The Journal of the Acoustical Society of America, 154(2), 1239–1247* .2023;10.1121/10.002076037615414

[2] : Clustering analysis of noise sources in healthcare facilities. *Applied Acoustics, 214, 109660* .2023;

[3] : A study on the conversion relationship of noise perceived annoyance and psychoacoustic annoyance—a case of substation noise. *Journal of Low Frequency Noise, Vibration and Active Control, 41(2), 810–818* .2022;

[4] : Noise assessment within healthcare facilities: annoyance and intrusiveness of sound sources. *—* .2025;

# Reviewer report: Reviewer response for version 2

**DOI:** 10.5256/f1000research.196838.r463111

**Published:** 2026-03-11

**Authors:** Azodo Adinife Patrick

**Affiliations:** 1 Department of Mechanical Engineering, Federal University Wukari, Wukari, Nigeria

- The revised title appears to lose some construct precision by replacing “acoustic comfort” with “comfort” and “noise annoyance” with “annoyance.” Since the manuscript consistently examines these outcomes specifically as acoustic comfort and noise annoyance, I suggest revising the title to: “The Effects of Hospital Noise and Noise Sensitivity on Patients’ Acoustic Comfort, Noise Annoyance, and Intention to Leave.” This would preserve clarity while remaining more closely aligned with the constructs analyzed in the paper.
- Table 1 still appears inconsistent with the explanation provided. In the current table, the category frequencies for Gender (112 + 114) and Marital Status (61 + 165) sum to 226, yet the corresponding Total rows are still reported as 205, while Hospital Ward is reported as 226. Since the manuscript also states that a total of 226 patients participated and that all analyses were conducted on the full sample, these Total entries appear to be residual table errors rather than missing responses. Please revise Table 1 so that all totals are reported consistently.
- Mediation-oriented wording still remains in the revised manuscript in places where no formal mediation analysis was conducted. For example, in the Figure 1 caption, the manuscript states that Noise Annoyance and Acoustic Comfort are “positioned as intermediate nodes in the probabilistic pathways from Lden and Sensitivity to Intention to Leave, consistent with a mediating structure.” In the Discussion, it further states that the pattern is “consistent with a mediating role for acoustic comfort,” and later describes the relationship as “a pathway suggestive of mediation.” Because mediation was not formally tested, I recommend replacing these phrases with more neutral language referring to intermediate or associated variables within the Bayesian network, rather than mediation.
- The rationale for the single-hospital setting is only partly reflected in the manuscript. The revised methods section now makes clear that the study was conducted in a public hospital in Saveh, Iran, which may be acceptable from a lenient review perspective. However, the fuller justification for why only one hospital was included is not explicitly inserted into the methods text itself.
- Minor copyediting issues remain. The manuscript still contains at least one obvious typographical error, such as “Forth” instead of “Fourth” in the limitations section, indicating that copyediting was not fully completed.

Is the work clearly and accurately presented and does it cite the current literature?

Partly

If applicable, is the statistical analysis and its interpretation appropriate?

Yes

Are all the source data underlying the results available to ensure full reproducibility?

Yes

Is the study design appropriate and is the work technically sound?

Yes

Are the conclusions drawn adequately supported by the results?

Partly

Are sufficient details of methods and analysis provided to allow replication by others?

Partly

Reviewer Expertise:

Industrial Engineering; Ergonomics; Health, safety,  and Environmental management

I confirm that I have read this submission and believe that I have an appropriate level of expertise to confirm that it is of an acceptable scientific standard, however I have significant reservations, as outlined above.

# Reviewer report: Reviewer response for version 2

**DOI:** 10.5256/f1000research.196838.r463112

**Published:** 2026-03-02

**Authors:** Simone Secchi, Elisa Nannipieri

**Affiliations:** 1 Universita degli Studi di Firenze (Ringgold ID: 9300), Florence, Tuscany, Italy

The paper has been significantly improved and is now suitable for publication

Is the work clearly and accurately presented and does it cite the current literature?

Partly

If applicable, is the statistical analysis and its interpretation appropriate?

I cannot comment. A qualified statistician is required.

Are all the source data underlying the results available to ensure full reproducibility?

Partly

Is the study design appropriate and is the work technically sound?

Partly

Are the conclusions drawn adequately supported by the results?

Yes

Are sufficient details of methods and analysis provided to allow replication by others?

Yes

Reviewer Expertise:

Environmental and room acoustics

We confirm that we have read this submission and believe that we have an appropriate level of expertise to confirm that it is of an acceptable scientific standard.

## References

[1] : Analysis of the Acoustic Comfort in Hospital: The Case of Maternity Rooms. *Buildings* .2022;12(8) : 10.3390/buildings12081117 10.3390/buildings12081117

# Reviewer report: Reviewer response for version 1

**DOI:** 10.5256/f1000research.185125.r452548

**Published:** 2026-02-09

**Authors:** Simone Secchi, Elisa Nannipieri

**Affiliations:** 1 Universita degli Studi di Firenze, Florence, Tuscany, Italy

The study shows that noise pollution in Iranian hospitals exceeds WHO standards. High noise levels and individual sensitivity reduce acoustic comfort, increasing discomfort and the desire to leave hospital early, thus hindering patient recovery.

 The article primarily uses the Bayesian Networks (BNs) model combined with delta-p sensitivity analysis to examine the relationships between the variables studied.

 to visualise and quantify how changes in one variable affect others.

 The study is interesting, but I think the following items should be clarified.

 In paragraph  2.3, it is unclear whether the measured level is an equivalent sound pressure level (i.e. an integration over the measurement period) or single instantaneous measurements.

 In the same paragraph please specify the model of the B&K sound level meter

 In my opinion, Table 2 is unclear. In the table, quantitative variables (such as Lden) are listed alongside qualitative variables (noise sensitivity, noise annoyance, etc.).

 I understand that 57.95 dBA is the Lden value, but I do not understand what 42.57 represents for noise sensitivity, and the same applies to the other qualitative variables.

 In Figure 1 and in the following please repalce Ldn with Lden

 Please control the percentages written in tables since the sums of different percentages in many cases is not 100%.

 In general, the correlation between the intention to leave and other environmental comfort variables other than noise should also be studied.

 Furthermore, the equivalent level alone is not a very meaningful parameter for assessing noise disturbance, since it would also have been useful to evaluate statistical levels such as L10 and L90.

Is the work clearly and accurately presented and does it cite the current literature?

Partly

If applicable, is the statistical analysis and its interpretation appropriate?

I cannot comment. A qualified statistician is required.

Are all the source data underlying the results available to ensure full reproducibility?

Partly

Is the study design appropriate and is the work technically sound?

Partly

Are the conclusions drawn adequately supported by the results?

Yes

Are sufficient details of methods and analysis provided to allow replication by others?

Yes

Reviewer Expertise:

Environmental and room acoustics

We confirm that we have read this submission and believe that we have an appropriate level of expertise to confirm that it is of an acceptable scientific standard, however we have significant reservations, as outlined above.

# Response

**Published:** 2026-02-18

**Authors:** Milad Abbasi

**Affiliations:** saveh university of medical sciences, Iran

**Response to Reviewer: Dr. Simone Secchi**

**Dear Dr. Secchi,**

 Thank you very much for your thoughtful and detailed review of our manuscript. Your comments have been invaluable in strengthening the clarity, methodological transparency, and overall quality of our work. Below, we provide a point-by-point response to each of your observations and concerns. All revisions have been made using track changes in the revised manuscript.

**Comment: **In paragraph 2.3, it is unclear whether the measured level is an equivalent sound pressure level (i.e. an integration over the measurement period) or single instantaneous measurements.

**Response to Comment 1:**

 Thank you for this important comment. We confirm that noise exposure was measured as equivalent continuous sound pressure levels (Leq) over consecutive 15-minute intervals, not as instantaneous spot measurements. The text has been revised accordingly to reflect this clarification.

**Comment: **In the same paragraph please specify the model of the B&K sound level meter

**Response to Comment 2:**

 Thank you for this technical comment. We have used B&K Sound Level Meter Analyzer, model 2250.

**Comment: **In my opinion, Table 2 is unclear. In the table, quantitative variables (such as Lden) are listed alongside qualitative variables (noise sensitivity, noise annoyance, etc.).

**Response to Comment 3:**

 Thank you for this important observation. You are correct that the original Table 2 presentation was unclear regarding the distinction between the original quantitative measurements and the categorized variables used in Bayesian Network modeling.

 We wish to clarify that all mentioned variables—including Lden, noise sensitivity, noise annoyance, acoustic comfort, and intention to leave—were originally measured as quantitative variables. The means and standard deviations reported in Table 2 represent the total measured mean and total measured SD for these continuous distributions across the entire sample. However, to run the Bayesian Network model and perform delta-p sensitivity analysis, these continuous variables were converted into ordinal qualitative variables (low, moderate, high) through dichotomization. This conversion is essential for instantiating discrete evidence scenarios (e.g., setting "Lden = High 100%") within the probabilistic graphical framework.

 Due to journal formatting restrictions on the number of tables, we were obligated to present both the continuous descriptive statistics and the categorical frequency distributions within a single table. To improve clarity and prevent misinterpretation, we have now revised the column headers in Table 2. The mean and standard deviation columns are now clearly labeled as:

**Total Measured Mean**

**Total Measured SD**

 This revision explicitly indicates that these values correspond to the original continuous measurements for each variable across all participants, while the frequency columns present the categorized distributions used in the Bayesian Network analysis.

**Comment: **I understand that 57.95 dBA is the Lden value, but I do not understand what 42.57 represents for noise sensitivity, and the same applies to the other qualitative variables.

**Response to Comment 4:**

 Thank you for this important observation. You are correct that the presentation of mean values in Table 2 requires clearer explanation. We confirm that all variables—including Lden, noise sensitivity, noise annoyance, acoustic comfort, and intention to leave—were originally measured as continuous quantitative variables. The mean and SD values reported in Table 2 represent the total measured mean and **total measured standard deviation for these continuous distributions across the entire sample, as follows:

 Lden: 57.95 dB (±6.61)

 Noise Sensitivity: 42.57 (±18.16)

 Noise Annoyance: 38.46 (±17.07)

 Acoustic Comfort: 4.73 (±1.45)

 Intention to Leave: 26.06 (±11.78)

 To improve clarity, we have revised Table 2 by:

 we Adding a clear explanatory footnote: “Mean and SD values represent the original continuous measurements for each variable prior to categorization.

**Comment: **In Figure 1 and in the following please repalce Ldn with Lden

**Response to Comment 5: **Thank you for pointing this out. We have corrected the notation and replaced “Ldn” with the correct “Lden” throughout Figure 1 and all subsequent occurrences in the manuscript (including figure captions, tables, and the main text) to ensure consistency.

**Comment: **Please control the percentages written in tables since the sums of different percentages in many cases is not 100%.

**Response to Comment 6: **Thank you for this careful observation. The percentages reported in the tables were originally presented with three decimal places, but the values had not been consistently rounded, which caused some rows not to sum exactly to 100% due to decimal precision effects.

 In the revised manuscript, the values were rounded correctly and consistently according to the original Bayesian Network outputs. As a result, the reported percentages now sum to 100% (1.000) in each probability distribution. This update only reflects proper rounding of the existing BN results, and no model outputs or analyses were changed.

**Comment: **In general, the correlation between the intention to leave and other environmental comfort variables other than noise should also be studied.

**Response to Comment 7:**

 Thank you for this thoughtful comment. We acknowledge that **intention to leave** is a complex, multi-dimensional construct that may be influenced by a range of environmental, clinical, and psychological factors beyond noise—including thermal comfort, lighting, indoor air quality, spatial layout, privacy, and overall satisfaction with the physical environment. The reviewer correctly notes that these variables were not included in our Bayesian Network model, and we agree this is an important consideration.

 However, we wish to clarify the scope and objective of the present study. This investigation was specifically designed to examine the isolated and interactive effects of acoustic environmental exposure (noise) and individual noise sensitivity on patient-reported outcomes. Our model was deliberately constructed to test theoretically grounded hypotheses regarding the direct and joint effects of these two primary variables, not to develop a comprehensive predictive model of all possible determinants of intention to leave.

 We have now explicitly acknowledged this limitation in Section 4.5:

 “Forth, this study focused exclusively on acoustic environmental factors and did not measure other physical environmental parameters (e.g., thermal comfort, lighting, air quality, privacy) that may also influence patients' intention to leave.”

**Comment: **Furthermore, the equivalent level alone is not a very meaningful parameter for assessing noise disturbance, since it would also have been useful to evaluate statistical levels such as L10 and L90.

**Response to Comment 8:**

 Thank you for this important methodological observation. We agree that Lden alone does not fully capture the temporal and spectral characteristics of the hospital acoustic environment that may contribute to patient disturbance. Statistical percentiles such as L10 and L90 provide valuable complementary information about noise variability and are widely used in environmental acoustics and psychoacoustics research.

 However, the primary objective of this study was to examine the relationship between overall noise exposure burden and patient-centered outcomes using a standardized, policy-relevant metric (Lden) that enables direct comparison with WHO guidelines and international studies. Lden is the most commonly reported metric in the hospital noise literature, facilitating cross-study comparability.

 Furthermore, due to our measurement protocol—which assessed noise exposure over consecutive 15-minute intervals within each three-hour block rather than through continuous 24-hour monitoring—we were not able to reliably compute statistical percentiles such as L10 and L90, as these require uninterrupted high-resolution time series data.

# Reviewer report: Reviewer response for version 1

**DOI:** 10.5256/f1000research.185125.r440738

**Published:** 2026-01-02

**Authors:** Azodo Adinife Patrick

**Affiliations:** 1 Department of Mechanical Engineering, Federal University Wukari, Wukari, Nigeria

**Reviewer Comments to the Author**

- The title is clear and informative but relatively long; consider whether it can be slightly shortened without loss of meaning.
- The abstract would benefit from explicitly stating the study design (cross-sectional) and the main analytical approach (Bayesian Network modeling).
- In the abstract, percentage changes are reported without clarifying how variables were categorized (low/moderate/high); brief clarification would improve readability.
- The introduction is comprehensive but lengthy; the final paragraphs could be streamlined to reduce repetition.
- The novelty of applying Bayesian Networks could be highlighted more explicitly as part of the research gap.
- The concept of “intention to leave” would benefit from clearer distinction from medical discharge or staff turnover.
- There is an inconsistency in reported sample size (205 vs. 226) across sections and tables that should be corrected.
- The rationale for selecting a single hospital setting should be briefly justified.
- The choice of Lden as the primary noise metric should be justified relative to other commonly used indicators (e.g., LAeq).
- It should be clarified whether noise measurements included weekdays, weekends, or both.
- The criteria used to categorize continuous variables into low, moderate, and high levels should be explicitly stated.
- The limitations of using a single-item VAS for intention to leave should be briefly acknowledged.
- The Bayesian Network structure development process (expert-driven, data-driven, or hybrid) should be clarified.
- A short, intuitive explanation of delta-p sensitivity analysis would improve accessibility for non-technical readers.
- The Results section contains some repetition between text, tables, and figures; selective summarization would improve clarity.
- Several tables are dense; clearer captions or brief interpretive notes would help guide the reader.
- Figure captions should be expanded to allow standalone interpretation.
- Some sentences in the Discussion are overly long and could be split for clarity.
- The mediating role of noise annoyance and acoustic comfort should be framed more cautiously, as mediation was not formally tested.
- Policy and design implications could be more clearly separated from theoretical discussion.
- The limitations section could be strengthened by acknowledging potential clinical confounders (e.g., pain, medication).
- The conclusion is appropriate but could be more concise and avoid repetition from the Discussion.
- Minor grammatical, typographical, and formatting inconsistencies are present and should be corrected through copyediting.

Is the work clearly and accurately presented and does it cite the current literature?

Partly

If applicable, is the statistical analysis and its interpretation appropriate?

Yes

Are all the source data underlying the results available to ensure full reproducibility?

Yes

Is the study design appropriate and is the work technically sound?

Yes

Are the conclusions drawn adequately supported by the results?

Partly

Are sufficient details of methods and analysis provided to allow replication by others?

Partly

Reviewer Expertise:

Industrial Engineering; Ergonomics; Health, safety,  and Environmental management

I confirm that I have read this submission and believe that I have an appropriate level of expertise to confirm that it is of an acceptable scientific standard, however I have significant reservations, as outlined above.

# Response

**Published:** 2026-02-18

**Authors:** Milad Abbasi

**Affiliations:** saveh university of medical sciences, Iran

**Response to Reviewer: Dr. Azodo Adinife Patrick**

**Dear Dr. Patrick,**

 Thank you very much for your thoughtful and detailed review of our manuscript. Your comments have been invaluable in strengthening the clarity, methodological transparency, and overall quality of our work. Below, we provide a point-by-point response to each of your observations and concerns. All revisions have been made using track changes in the revised manuscript.

**Comment: **The title is clear and informative but relatively long; consider whether it can be slightly shortened without loss of meaning.

**Response to Comment 1: **The Effects of Hospital Noise and Noise Sensitivity on Patient`s Comfort, Annoyance, and Intention to Leave

**Comment: **The abstract would benefit from explicitly stating the study design (cross-sectional) and the main analytical approach (Bayesian Network modeling).

**Response to Comment 2: **Thank you for this important methodological observation.

**Comment: **In the abstract, percentage changes are reported without clarifying how variables were categorized (low/moderate/high); brief clarification would improve readability.

**Response to Comment 3: **Thank you for this helpful comment. We agree that reporting percentage changes in the abstract without briefly indicating how “low/moderate/high” categories were defined may reduce readability. In the revised abstract, we have added a short clarification stating that continuous variables were categorized using percentile-based thresholds: low (P75). This concise note improves interpretability while keeping the abstract within the word limit.

**Comment: **The introduction is comprehensive but lengthy; the final paragraphs could be streamlined to reduce repetition.

**Response to Comment 4: **The introduction was revise according to this comment

**Comment: **The novelty of applying Bayesian Networks could be highlighted more explicitly as part of the research gap.

**Response to Comment 5: **The introduction was revise according to this comment

**Comment: **There is an inconsistency in reported sample size (205 vs. 226) across sections and tables that should be corrected.

**Response to Comment 6: **Thank you for this comment. I clarify the sample size reporting as follows:

 “The minimum required sample size was determined using G*Power 3.1 software, assuming a medium effect size (0.3), α = 0.05, and a statistical power of 0.95. Based on these parameters, a minimum sample of 178 participants was deemed necessary. To account for potential attrition, this number was increased by 15%, yielding a final sample of 205 participants —the minimum acceptable number for sufficient statistical power and adequate generalizability. During data collection, a total of 226 eligible patients were recruited and participated voluntarily. This larger achieved sample strengthens the generalizability of the findings and is fully consistent with the sampling strategy, as no upper limit was imposed on participation.”

 Importantly, the Methods section (Section 2.2) clearly states that 205 was the target sample size and that this number was increased from the minimum required 178 to account for attrition. The text does not state that 205 was a fixed maximum, nor does it preclude the inclusion of additional participants. The achieved sample of 226 is therefore fully consistent with the stated sampling strategy. All analyses were conducted on the full sample of 226 participants. Tables and frequency distributions have been verified and corrected to consistently reflect N = 226 throughout the manuscript.

**Comment: **The rationale for selecting a single hospital setting should be briefly justified.

**Response to Comment 7: **Thank you for this comment This study was conducted at a public hospital in Saveh, Iran. Although Saveh has three hospitals, two are affiliated with the private sector and, due to their internal policies, were unable to participate in this research. Accordingly, data collection was necessarily limited to the single hospital that granted approval. For ethical reasons, the names of the institutions that declined participation are not disclosed.

**Comment: **The choice of Lden as the primary noise metric should be justified relative to other commonly used indicators (e.g., LAeq).

**Response to Comment 8:** Thank you for your comment. We selected Lden (day-evening-night weighted equivalent level) as our primary noise metric because it applies empirically derived +5 dB (evening) and +10 dB (night) penalties that reflect increased human sensitivity during rest periods—a critical consideration in hospital settings where sleep disruption directly impacts patient recovery. Unlike unweighted LAeq, Lden is the standard indicator recommended by the World Health Organization and the European Environmental Noise Directive for health-based noise impact assessment, ensuring policy relevance and comparability across studies. Furthermore, Lden is explicitly calibrated to exposure-response relationships for annoyance and sleep disturbance, our primary outcomes of interest. Thus, Lden was not a default choice but a deliberate selection aligned with our research questions, regulatory context, and the unique temporal sensitivities of the hospital acoustic environment.

**Comment: **It should be clarified whether noise measurements included weekdays, weekends, or both.

**Response to comment 9:** Thank you for this important methodological clarification. We confirm that the noise measurements included weekdays and weekends.

 Measurements were conducted over a 12-week period from November 2024 to February 2025, with a full 24-hour measurement cycle per week in each participating ward. These cycles were systematically rotated across weekdays to ensure that the acoustic conditions were representative of both weekdays (Saturday to Thursday in Iran) and weekends (Friday). This approach was necessary because hospital activity patterns—including staffing levels, visitor attendance, non-emergency procedures, and patient movements—differ significantly between weekdays and weekends, and measurement exclusively on weekdays would overestimate the true level of noise exposure.

**Comment: **The criteria used to categorize continuous variables into low, moderate, and high levels should be explicitly stated.

**Response to comment 10:** Thank you for this important comment. We agree that the criteria used for categorizing continuous variables should be clearly reported. In the revised manuscript, we now explicitly state that continuous variables were discretized using sample-based percentile thresholds. Specifically, values below the 25th percentile (P25) were classified as *low*, values between the 25th and 75th percentiles (P25–P75) were classified as *moderate*, and values above the 75th percentile (P75) were classified as *high*. These thresholds were calculated from the distribution of the study data and were applied consistently across all variables used in the Bayesian Network analysis.

**Comment: **The limitations of using a single-item VAS for intention to leave should be briefly acknowledged.

**Response to comment 11:** While thanking you for this valuable comment, this was added to the limitations of the study.

**Comment: **The Bayesian Network structure development process (expert-driven, data-driven, or hybrid) should be clarified.

**Response to comment 12:** Thank you for this comment. We agree that the Bayesian Network (BN) structure development process should be clarified. In the revised manuscript, we explain that the BN topology (nodes and directed links) was constructed based on the study structure and the variables/domains captured by the questionnaires used in this study. The purpose of using the BN was not to “predict” the structure, but to quantify the interdependencies among nodes and to evaluate how changes in the state of one or more nodes (evidence instantiation) update the probability distributions of other variables (probabilistic inference / evidence propagation). Accordingly, the conditional probability tables (CPTs) were estimated from the observed study data using GeNIe.

**Comment: **A short, intuitive explanation of delta-p sensitivity analysis would improve accessibility for non-technical readers.

**Response to comment 13: **Thank you for this helpful suggestion. We agree that a short, intuitive explanation of Δp sensitivity analysis would improve accessibility for non-technical readers. In the revised manuscript, we added a brief clarification explaining that Δp represents the change in probability of each state of a target node (low/moderate/high) after setting evidence on an input node to a specific state (low/moderate/high). We also clarified that Δp is computed as updated probability minus baseline probability for each target state, and that larger |Δp| indicates a stronger influence, while the sign indicates an increase or decrease in that target-state probability.

**Comment: **The Results section contains some repetition between text, tables, and figures; selective summarization would improve clarity.

**Response to comment 14:** Thank you for your valuable comment. We have deliberately summarized the descriptive findings to maintain conciseness. Regarding the Bayesian analysis, given that not all readers may be fully familiar with this methodological approach and its interpretation, we aimed to present only the essential interpretations—including one detailed example—while avoiding excessive elaboration. A comprehensive interpretation of all results would have substantially increased the length of the manuscript without proportional benefit to clarity.

**Comment: **Several tables are dense; clearer captions or brief interpretive notes would help guide the reader.

**Response to Comment 15: **Thank you for this excellent practical suggestion. The brief interpretive notes were added.

**Comment: **Figure captions should be expanded to allow standalone interpretation.

**Response to Comment 16: **Thank you for this excellent practical suggestion. The brief interpretive captions were added.

**Comment: **Some sentences in the Discussion are overly long and could be split for clarity.

**Response to Comment 17: **Thank you for this important observation regarding readability. We have conducted a careful, line-by-line review of the entire Discussion section and have revised according to this comment.

**Comment: **The mediating role of noise annoyance and acoustic comfort should be framed more cautiously, as mediation was not formally tested.

**Response to Comment 18:**

 Thank you for this methodologically rigorous and important observation. You are absolutely correct. While our Bayesian Network model **implies** mediation by positioning noise annoyance and acoustic comfort as intermediate nodes between Lden/sensitivity and intention to leave, we did not conduct formal mediation analysis (e.g., bootstrap tests of indirect effects, Bayesian mediation with credible intervals, or counterfactual-based approaches). Our use of terms such as "mediator," "mediating role," and "mediating factor" throughout the Discussion and Conclusion overstates the evidentiary support for mediation given our analytical approach.

 We have revised the manuscript to replace all claims of mediation with more cautious language that accurately reflects what our Bayesian Network demonstrates: probabilistic dependence structures consistent with mediation, not confirmed causal pathways.

**Comment: **Policy and design implications could be more clearly separated from theoretical discussion.

**Response to Comment 19:**

 Thank you for your valuable comment. In accordance with your request, all sentences and phrases related to policy and design implications that were scattered throughout the Discussion section have been extracted and consolidated into one dedicated and focused subsection titled "6. Policy and Design Implications **"**.

**Comment: **The limitations section could be strengthened by acknowledging potential clinical confounders (e.g., pain, medication).

**Response to Comment 20:**

 Thank you for this important methodological observation. These confounders were mentioned as follows:

 “Third, although ward type was accounted for, other potentially influential clinical factors—such as pain levels, medication use, or individual health conditions—were not controlled. These unmeasured confounders may have influenced patients' noise perceptions and tolerance.”

**Comment: **The conclusion is appropriate but could be more concise and avoid repetition from the Discussion.

**Response to Comment 21:**

 Thank you for this important observation. The conclusion was revised as follows:

 Hospital soundscapes are active components of the care environment, not merely ambient features. This study demonstrates that noise exposure and individual noise sensitivity synergistically increase annoyance, reduce acoustic comfort, and elevate patients' intention to leave. The findings position acoustic comfort and noise annoyance as intermediate variables in these pathways. Addressing noise pollution—particularly through targeted interventions for noise-sensitive individuals and improved acoustic design—is therefore an essential dimension of quality healthcare delivery.

**Comment: **Minor grammatical, typographical, and formatting inconsistencies are present and should be corrected through copyediting.

**Response to Comment 21:**

 Thank you for this important observation. We have conducted a thorough, line-by-line copyediting review of the entire manuscript. All identified errors have been corrected.
